# Gelatin-Based Hydrogels for Peripheral Nerve Regeneration: A Multifunctional Vehicle for Cellular, Molecular, and Pharmacological Therapy

**DOI:** 10.3390/gels11070490

**Published:** 2025-06-25

**Authors:** Denisa-Madalina Viezuina, Irina Musa, Madalina Aldea, Irina-Mihaela Matache, Alexandra-Daniela Rotaru Zavaleanu, Andrei Gresita, Sfredel Veronica, Smaranda Ioana Mitran

**Affiliations:** 1 Doctoral School, University of Medicine and Pharmacy Craiova, 200349 Craiova, Romania; viezuina.denisa@gmail.com (D.-M.V.);; 2 Department of Psychiatry, University of Medicine and Pharmacy of Craiova, 200349 Craiova, Romania; 3 Faculty of Medicine, Carol Davila University of Medicine and Pharmacy, 050474 Bucharest, Romania; irina.m.matache@gmail.com; 4 Department of Epidemiology, University of Medicine and Pharmacy of Craiova, 2-4 Petru Rares Str., 200349 Craiova, Romania; alexandra.rotaru@umfcv.ro; 5 Experimental Research Centre for Normal and Pathological Aging, University of Medicine and Pharmacy of Craiova, 200349 Craiova, Romania; andrei.gresita@umfcv.ro (A.G.); smaranda.mitran@umfcv.ro (S.I.M.); 6 Department of Physiology, University of Medicine and Pharmacy of Craiova, 2-4 Petru Rares Str., 200349 Craiova, Romania

**Keywords:** peripheral nerve regeneration, gelatin-based hydrogels, controlled drug delivery, neurotrophic factors, neuroprotection

## Abstract

Peripheral nerve injuries (PNIs) present a significant clinical challenge due to the inherently limited regenerative capacity of the adult nervous system. Conventional therapeutic strategies, such as nerve autografting and systemic pharmacological interventions, are often limited by donor site morbidity, restricted graft availability, and suboptimal drug bioavailability. In this context, gelatin-based hydrogels have emerged as a promising class of biomaterials due to their excellent biocompatibility, biodegradability, and structural similarity to the native extracellular matrix. These hydrogels could offer a highly tunable platform capable of supporting cellular adhesion, promoting axonal elongation, and enabling localized and sustained release of therapeutic agents. This narrative review synthesizes recent advances in the application of gelatin-based hydrogels for peripheral nerve regeneration, with a particular focus on their use as delivery vehicles for neurotrophic factors, stem cells, and pharmacologically active compounds. Additionally, this review provides a foundation for extending our ongoing preclinical study, evaluating the neuroregenerative effects of alpha-lipoic acid, B-complex vitamins, and a deproteinized hemoderivative in a murine PNI model. Although systemic administration has demonstrated promising neuroprotective effects, limitations related to local drug availability and off-target exposure highlight the need for site-specific delivery strategies. In this regard, gelatin hydrogels might represent an excellent candidate for localized, controlled drug delivery. The review concludes by discussing formulation techniques, manufacturing considerations, biological performance, and key translational and regulatory aspects.

## 1. Background

Peripheral nerve injuries (PNIs) pose significant clinical challenges due to the limited intrinsic regenerative capacity of peripheral nerves and the complex microenvironment required for successful repair. One major obstacle is the slow and often incomplete axonal regeneration, which typically occurs at a rate of 1–3 mm per day [1]. This can lead to prolonged periods of denervation, during which target organs such as muscles may undergo irreversible atrophy and fibrosis, severely compromising functional recovery [2]. Moreover, even when axons do regenerate, there is a high risk of misdirected reinnervation, where motor axons may wrongly connect to sensory pathways and vice versa, resulting in poor functional outcomes [3]. The formation of neuromas and scar tissue at the injury site can further impede regeneration by creating physical and biochemical barriers [4]. Clinically, patients with PNIs often experience a range of long-term deficits. Motor impairments may include muscle weakness, paralysis, or impaired coordination, depending on the severity and location of the injury. Sensory deficits commonly involve numbness, paresthesia (tingling or burning sensations), allodynia (pain caused by non-painful stimuli), or complete loss of sensation in the affected area [5]. These deficits can significantly reduce quality of life, hinder daily functioning, and represent a persistent therapeutic challenge [6].

The currently available treatment options for peripheral nerve injuries include direct end-to-end suturing, autologous nerve grafting, and the use of nerve guidance conduits [7]. Direct suturing is feasible only in cases where tension-free approximation of nerve ends is possible, which is rare in larger defects [8]. Notably, autologous nerve grafts remain the gold standard for bridging nerve gaps [9]. However, they present several limitations, such as limited donor tissue availability, mismatch in fascicular architecture, and the risk of donor site morbidity, including neuroma formation and functional loss at the harvesting site [10]. Thus, despite advances current treatments are often inadequate for achieving full functional recovery in severe nerve injuries.

Consequently, studies based on tissue engineering techniques have become popular, especially the use of nerve guidance conduits enhanced with bioactive hydrogels aiming to replicate the native extracellular matrix (ECM) and support axonal regrowth [11,12]. For instance, collagen, the main structural protein in native ECM, is a widely used material that can support Schwann cell migration and axon elongation [13]. Chitosan, derived from chitin, exhibits favorable biodegradability and antimicrobial properties and has been used in both pure and blended forms with gelatin or collagen to enhance nerve repair [14]. Hyaluronic acid (HA) hydrogels, often chemically modified for stability, provide a hydrophilic, ECM-mimicking environment that facilitates nutrient diffusion and cellular infiltration [15]. Fibrin hydrogels, naturally formed from fibrinogen and thrombin, have also shown promise due to their rapid gelation and intrinsic bioactivity [16]. More advanced approaches incorporate composite hydrogels, such as gelatin–silk fibroin or gelatin–polycaprolactone blends, to combine the biological activity of natural polymers with the mechanical strength and processability of synthetic ones [17].

Interestingly, the denatured derivative of collagen, gelatin can be physically crosslinked to improve mechanical properties, degradation rates, and loading capacity for cells or growth factors (e.g., by methacrylation to generate GelMA) [18]. Due to their biocompatibility, biodegradability, and natural bioactivity, gelatin-based hydrogels have emerged as promising candidates among biomaterials [19]. Gelatin methacryloyl is one of the most extensively studied gelatin-based hydrogels due to its tunable mechanical properties, crosslink properties, and retention of bioactive motifs such as arginine-glycine-aspartic acid (RGD) peptides, which can promote cell adhesion and axonal growth [18]. These features make gelatin hydrogels very flexible in delivering biological signals and supporting cellular development [20]. Notably, advances in gelatin-based hydrogels, including 3D printing, growth factor loading, conductive enhancements, and stem cell encapsulation, have led to multifunctional nerve guidance conduits that could meet both structural and biological demands of nerve regeneration [21]. In vivo models, studies have shown that such hydrogels not only offer mechanical support but also help axon elongation, Schwann cell migration, angiogenesis, and functional recovery [22,23].

This article is a narrative review that provides a comprehensive synthesis of recent advances in the application of hydrogels, particularly gelatin-based, for peripheral nerve regeneration. It critically examines formulation techniques, fabrication strategies, experimental models, and therapeutic outcomes, highlighting the emerging role of gelatin hydrogels in regenerative neuroscience. In parallel, the review establishes a conceptual and methodological framework that supports the future development of our ongoing preclinical research in a murine model of peripheral nerve injury. In this study, we investigate the systemic administration of a deproteinized hemoderivative of calf blood and alpha-lipoic acid in combination with B-complex vitamins, which has shown preliminary neuroprotective and neuroregenerative potential. However, given the limitations of systemic delivery, such as low bioavailability and insufficient site-specific activity we analyze the potential of gelatin hydrogels as localized, controlled delivery vehicles. Ultimately, we discuss limitations such as the potential for clinical translation and regulatory considerations.

**Methodology**: For this narrative review, a literature search was conducted using the PubMed database to identify relevant studies published between 2018 and 2025. The search strategy employed the free-text terms: “peripheral nerve”, “hydrogels”, and “gelatin”. The search was restricted to English-language articles and focused on studies that explored the use of gelatin-containing hydrogels in the context of peripheral nerve regeneration. The final selection included both in vitro and in vivo studies that provided detailed descriptions of hydrogel formulation, experimental design, and outcomes related to nerve repair.

## 2. Gelatin Hydrogel Fabrication and Functionalization Techniques

**Figure 1:**
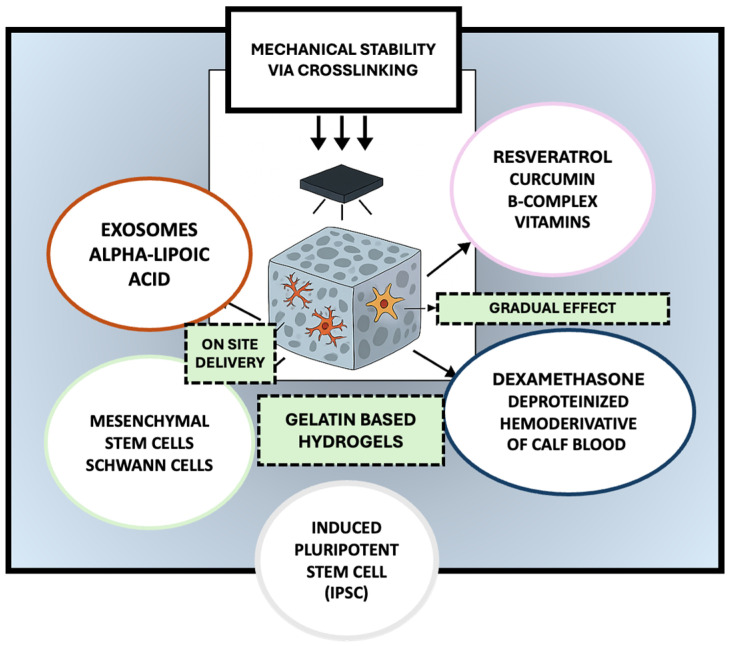
Multifunctional gelatin-based hydrogels for peripheral nerve regeneration: a platform for cellular support, bioactive delivery, and tissue repair. The selected artwork was taken or adapted from pictures provided by Servier Medical Art (Servier; https://smart.servier.com/, accessed on 13 March 2025), licensed under a Creative Commons Attribution 4.0 Unported License [33].

Due to their inherent biocompatibility, biodegradability, and structural resemblance to the native extracellular matrix, gelatin-based hydrogels have garnered considerable attention as scaffolding materials in peripheral nerve regeneration, as they could provide a supportive environment for axonal regrowth and cellular infiltration while enabling the localized delivery of therapeutic agents [24]. However, native gelatin hydrogels often suffer from limited mechanical strength and rapid degradation, which can impede their applicability [25]. To address these limitations, recent research has focused on modifying and functionalizing gelatin hydrogels to enhance their mechanical stability, bioactivity, and integration with host tissue [26]. One widely adopted strategy involves the chemical modification of gelatin with methacrylate groups to produce gelatin methacryloyl, a photocrosslinkable hydrogel that allows for precise tuning of stiffness and degradation rate through light-mediated polymerization [27]. GelMA has demonstrated improved structural integrity and has been shown to support Schwann cell adhesion and proliferation [28]. Additionally, composite hydrogels combining gelatin with other natural or synthetic polymers have been explored to reinforce mechanical performance. For example, gelatin–chitosan and gelatin–silk fibroin composites exhibit enhanced tensile strength and slower degradation while maintaining favorable biological properties [29]. Functionalization with bioactive molecules further enhances the regenerative potential of gelatin-based hydrogels. Incorporation of RGD motifs, which promote integrin-mediated cell adhesion, and matrix metalloproteinase (MMP)-sensitive linkers, which enable cell-mediated remodeling, has been shown to improve host–scaffold interactions [30]. Moreover, gelatin hydrogels have been employed as delivery platforms for neurotrophic factors such as nerve growth factor and brain-derived neurotrophic factor (BDNF) [31], as well as pharmacological agents including curcumin, tacrolimus (FK506), methylcobalamin [32], alpha-lipoic acid, deproteinized hemoderivative of calf blood, and B-complex vitamins (Figure 1).

Gelatin hydrogels interact with encapsulated agents, such as BDNF and tacrolimus through a combination of electrostatic interactions, hydrogen bonding, and physical entrapment within the crosslinked polymeric network [34]. The cationic regions of gelatin, derived from its amino acid residues (e.g., lysine and arginine), exhibit high affinity for anionic biomolecules such as BDNF, facilitating non-covalent binding that stabilizes the protein structure and mitigates proteolytic degradation [35]. This interaction not only prolongs bioavailability but also enables sustained, localized release, which is essential for activating tropomyosin receptor kinase B (TrkB) receptors on regenerating axons and promoting Schwann cell survival and migration [36]. For hydrophobic small molecules like tacrolimus, encapsulation is typically achieved via hydrophobic domain partitioning or micellar inclusion within modified gelatin matrices [37]. Tacrolimus interacts with FK506-binding proteins (FKBPs) to inhibit calcineurin, which could result in enhanced neurite outgrowth through upregulation of growth-associated genes such as GAP-43 and βIII-tubulin [37]. Notably, gelatin’s porous microarchitecture could support nutrient diffusion and axonal infiltration while gradually degrading in vivo, ensuring temporal coordination between scaffold support and drug release [38]. The following sections will explore in greater detail the fabrication techniques and functional modifications that mark their growing relevance in peripheral nerve repair.

## 3. Evaluation of Gelatin-Based Hydrogels for Peripheral Nerve Regeneration

### 3.1. Comparative Analysis of Gelatin-Based Hydrogels vs. Other Natural and Synthetic Hydrogels

Gelatin-based hydrogels offer a unique balance between biological fidelity and tunability in comparison to other widely studied hydrogel systems such as alginate, chitosan, and polyethylene glycol (PEG)-based matrices. Gelatin, derived from denatured collagen, contains inherent bioactive motifs, such as arginine-glycine-aspartic acid (RGD) sequences and matrix metalloproteinase (MMP)-sensitive domains, that actively support cell adhesion, migration, and enzymatic remodeling. This biological responsiveness is often absent in alginate and PEG systems unless they are chemically modified. For instance, native alginate hydrogels, while structurally robust and ionically crosslinkable via divalent cations (e.g., Ca^2+^), lack integrin-binding sites and typically require conjugation with adhesion peptides to become biointeractive. In contrast, gelatin hydrogels exhibit spontaneous cell–matrix interactions without exogenous modifications. From a degradation and immunogenicity standpoint, chitosan hydrogels share some advantages over gelatin due to their natural origin and mild degradation profile. However, chitosan’s cationic charge and poor solubility at physiological pH can restrict uniform encapsulation of certain proteins or drugs and provoke low-grade inflammation. Gelatin, especially when methacrylated, permits photocrosslinking under mild conditions and displays a more predictable degradation rate. PEG-based hydrogels, while highly tunable and non-immunogenic, are biologically inert unless functionalized, and they often serve as structural carriers rather than bioactive matrices. Moreover, unlike gelatin or chitosan, PEG hydrogels can inhibit cell infiltration due to their dense, hydrophilic polymer network, unless modified with degradable linkers or porogenic agents [14,39,40,41] (Table 1).

### 3.2. Basic and Functionalized GelMA Hydrogels

One of the most widely adopted strategies to improve the mechanical stability, bioactivity, and host tissue integration of gelatin is its chemical modification via methacrylation, resulting in the formation of GelMA [27]. Under UV or visible light, this derivative allows photo-crosslinking under photoinitiators such as Irgacure 2959 or lithium phenyl-2,4,6-trimethylbenzoylphosphinate (LAP), so enabling the formation of hydrogels with varying stiffness, porosity, and degradation profile [42]. GelMA has been shown to support Schwann cell viability and axonal regrowth in peripheral nerve repair models [28]. To further improve the mechanical and biological characteristics of gelatin hydrogels, researchers have incorporated additional polymers. For instance, researchers combined GelMA with silk fibroin methacrylate (SF-MA) to form a double-crosslinked hydrogel with enhanced β-sheet crystallinity and elasticity [43]. Similarly, others integrated polyethylene glycol diacrylate (PEGDA) into GelMA matrices to fabricate 3D-printed, multichannel conduits with favorable viscoelastic properties for guiding axonal regeneration [44]. Soucy et al. (2018) introduced tropoelastin into GelMA to develop an adhesive hydrogel with superior elasticity and tissue bonding strength, promoting “sutureless” nerve repair [45]. Different crosslinking strategies were also employed depending on the application. Photocrosslinking was predominant due to its speed and spatial precision. In contrast, enzymatic systems like horseradish peroxidase (HRP)/H_2_O_2_ were used to form tyramine-substituted hyaluronic acid microparticles embedded in gelatin hydrogels [46]. In earlier designs, glutaraldehyde was used to chemically stabilize gelatin conduits, providing the mechanical rigidity required for implantation [47].

A representative example of a multifunctional GelMA-based hydrogel system is presented in a study by Luo et al. who developed a gelatin methacryloyl hydrogel with basic fibroblast growth factor and DPSC. The hydrogels, which were synthesized using a reaction of gelatin with methacrylic anhydride, were combined with basic Fibroblast Growth Factor (bFGF) and photoinitiator Irgacure 2959, and photocrosslinked under UV light. Three concentrations of GelMA were tested to optimize mechanical and biological performance. The hydrogels showed a highly porous and interconnected structure, with varying swelling capacity and increasing mechanical strength. Spectroscopic analyses confirmed the preservation of essential biological motifs, such as RGD sequences and MMP-degradable sites, crucial for cell adhesion and remodeling. The degradation rate and bFGF release kinetics varied with GelMA concentration, indicating its capacity for sustained molecular delivery. In vitro experiments showed that the gels supported DPSC viability and proliferation. Additionally, an in vivo model was established using a 15-mm sciatic nerve defect in rats, demonstrating its potential as a multifunctional platform for neural tissue engineering [48]. Sukhinich et al. (2020) also explored the use of gelatin-based conduits for peripheral nerve repair [49]. The gelatin hydrogel, prepared by mixing porcine gelatin with glutaraldehyde, was used in an in vivo mouse study to bridge sciatic nerve gaps. The conduits, which had a 0.8 mm lumen and 0.4 mm wall thickness, supported tissue integration and provided a favorable scaffold for cell adhesion and migration without additional functionalization. The study found that the conduits facilitated nerve fiber regrowth and Schwann cell migration, with the cortex group showing significant motor function improvement by week 8. However, muscle atrophy was not reversed. The study highlights the potential of gelatin-based conduits for nerve regeneration and the potential of these conduits in treating peripheral nerve injuries [49].

### 3.3. Gelatin Hydrogels for Delivery of Drugs, Cells, and Growth Factors

In addition to providing structural support hydrogels have been extensively explored across diverse biomedical applications as versatile delivery systems, demonstrating their capacity to encapsulate and release bioactive compounds in a controlled manner. For instance, dual-sensitive hydrogels, such as a thermosensitive poly (N-isopropyl acrylamide)-co-acrylic acid (NIPAM-co-AA/TA) system loaded with curcumin, have shown on-demand antibacterial drug release in response to wound-specific pH and temperature changes, highlighting their therapeutic precision and versatility [50]. Similarly, naturally derived hydrogels combining keratin, protocatechuic aldehyde, and iron ions have exhibited injectability, self-healing, tissue adhesion, and anti-inflammatory capabilities, significantly promoting diabetic wound healing via macrophage modulation [51]. Beyond wound care, supramolecular D-peptide hydrogels have also been successfully employed in gastrointestinal applications. A novel dextrorotary (D)-peptide supramolecular hydrogel (CP-CNDS) has been developed to treat postoperative pancreatic fistula by selectively capturing and sequestering digestive enzymes such as trypsin, chymotrypsin, and lipase within its gel matrix. This entrapment effectively suppresses enzyme activity and prevents further tissue damage [52].

Such systems have also been adapted for use in peripheral nerve models. Research approaches incorporated growth factors such as VEGF, NGF, and bFGF into GelMA matrices, resulting in sustained release profiles that facilitated angiogenesis, neurite outgrowth, and cell proliferation [53]. To promote neuroregeneration, some researchers have employed 7,8-dihydroxyflavone, a selective TrkB receptor agonist, shown to enhance axonal outgrowth and neuronal survival. Others have targeted the immune microenvironment by incorporating anti-inflammatory compounds such as proanthocyanidins and dexamethasone into biomaterial systems, effectively reducing oxidative stress and modulating immune responses to support tissue regeneration [54]. Many of these hydrogel systems also supported cell-based therapies. Schwann cells, neural stem cells and olfactory stem cells were encapsulated in gelatin matrices to enhance axonal regeneration, remyelination, and neurotrophic support. For example, Zhou et al. (2018) demonstrated that neural stem cells (NSCs) delivered in gelatin hydrogels significantly improved myelination and functional recovery in a sciatic nerve model [55], while Esaki et al. (2019) reported improved regeneration following olfactory stem cells (OSC) transplantation in a facial nerve injury model [56]. Aregueta-Robles et colleagues showed that co-culturing Schwann cells and PC12 cells within gelatin–PVA hydrogels supported the formation of 3D neural networks and extensive neurite outgrowth [57].

In another study, Zhang et al. (2023) focused on the use of a gelatin methacrylate hydrogel system for the sustained delivery of VEGF and NGF, combined with custom chitin conduits for peripheral nerve repair in rats [58]. The hydrogel was prepared by dissolving glycidyl methacrylate (GM) with a photoinitiator, adding VEGF and NGF for bioactivity, and applying UV light for crosslinking (CT/GM/Gf group). The structure showed porous morphology, ideal for nutrient exchange, and confirmed sustained growth factor release over 14 days. It promoted cell proliferation, angiogenesis, and migration of Human umbilical vein endothelial cells (HUVECs), and reduced apoptosis in PC-12 neuronal cells. The study included both in vitro and in vivo approaches using a rat nerve transposition model with a 16-week follow-up. In vivo, the CT/GM/GF group showed the highest compound muscle action potentials, reduced muscle atrophy, enhanced axon diameter, myelin thickness, motor/sensory neuron recovery, and superior functional motor recovery compared to control and single-growth-factor groups [58].

Furthermore, Xu et al. (2022) have developed a VEGF-loaded gelatin-based hydrogel for peripheral nerve repair after crush injury [20]. The hydrogel is based on gelatin modified by methacrylic anhydride, dissolved in a lithium phenyl-2,4,6-trimethylbenzoylphosphinate (LAP) solution. VEGF165 is incorporated, and the mixture is crosslinked under 405 nm UV light to form VEGF@GelMA hydrogels. The hydrogel has a porous microstructure for nutrient diffusion and cell infiltration, and sustained VEGF release over several days. The study was conducted in vitro using RSC96 Schwann cells and HUVECs for cell viability, morphology, and angiogenesis, and in vivo using a rat sciatic nerve crush injury model. The results showed improved functional recovery, enhanced axon regeneration and remyelination, significant angiogenesis, reduced muscle atrophy, and greater muscle fiber area in treated rats. This injectable GelMA hydrogel with controlled VEGF delivery might show promise for minimally invasive peripheral nerve repair strategies [20].

Focusing on the therapeutic versatility of gelatin-based systems, Li et al. (2023) have developed a GelMA-based hydrogel system incorporating CGRP (calcitonin gene-related peptide) to promote diabetic wound healing [59]. The hydrogel was prepared by dissolving 10% gelatin methacrylate in solution with lithium phenyl-2,4,6-trimethylbenzoylphosphinate (as a photoinitiator. CGRP was added at 2 µg/mL and photo-crosslinked under 405 nm UV light for 10 s to form the GelMA-CGRP hydrogel. The hydrogel showed a porous structure, sustained release of CGRP for at least 14 days, improved mechanical properties, strong antioxidant and antibacterial activity, and maintained biocompatibility with high cell viability in L929 fibroblasts. The study was conducted in vitro using bone marrow macrophages, HUVECs, and dorsal root ganglia, and in vivo using a Type 2 diabetic mouse wound model. The results showed that the GelMA-CGRP hydrogel accelerated wound closure by improving collagen deposition, re-epithelialization, and angiogenesis. Also, it promoted M2 macrophage polarization, enhanced endothelial proliferation, migration, and VEGF secretion, and outperformed GelMA-only controls in reducing inflammation and oxidative stress in diabetic wounds [59]. Complementing these findings, Javanmardi presents an injectable drug-delivery hydrogel system for peripheral nerve repair, involving a combination of proanthocyanidin (PA), hyaluronic acid microparticles, and dexamethasone (Dex). The hydrogel has high compressive strength, improved biodegradability, and sustained Dex release for up to 18 days. It is porous, injectable, and cytocompatible. The primary drug release mechanism is Fickian diffusion. The study was conducted in vitro using adipose-derived stem cell encapsulation and in vivo using a rat sciatic nerve crush injury model. The results showed that the gel-PA/Dex-Mp hydrogel had the highest spine functional index SFI score improvement and reduced sensory deficit. It also showed enhanced axon regeneration, myelin integrity, and minimal Vacuolization. The hydrogel is biocompatible and superior to either Dex-Mp or Gela-PA alone, demonstrating excellent potential for controlled drug delivery and peripheral nerve regeneration [60].

### 3.4. Conductive and Piezoelectric Hydrogels

Another branch of research has incorporated electrical functionality into gelatin hydrogels. In a recent study, Javidi et al. (2023) present a novel gelatin-based hydrogel for neural tissue engineering, focusing on enhancing neuronal differentiation through conductivity and piezoelectric effects [61]. The conduit has a core–shell structure, with a shell made of polycaprolactone (PCL)/polyvinylidene fluoride (PVDF) with gelatin and 2% polyaniline/graphene (PAG) nanocomposite. The core is filled with chitosan–gelatin hydrogel containing PAG and ZnO nanoparticles, crosslinked with glutaraldehyde and freeze-dried (lyophilized). The hybrid scaffold has nanofibrous morphology, hydrophilic surfaces, enhanced mechanical strength, biodegradability, and electrical conductivity. The piezoelectric output voltage improved from 400 mV to 1000 mV with PAG. The study used PC12 cells cultured on the conduits, showing improved adhesion, proliferation, and neuronal differentiation. PAG incorporation also improved antibacterial activity against *E. coli* and *S. aureus* [61]. Expanding on scaffold electroactivity, Neuman explores the impact of electrical and magnetic stimulation on neurite outgrowth in gelatin-based hydrogels [62]. Researchers developed two hydrogels: GelMA and Gel-Amin, an ionically conductive hydrogel incorporating choline acrylate. Gel-Amin mimics the ionic conductivity of natural nerve tissue and is optimized to mechanically match the elasticity of native nerve. It has a 4x greater ionic conductivity than GelMA, higher elastic moduli, and higher swelling capacity. The study involved encapsulating neonatal rat dorsal root ganglia and Schwann cells in each hydrogel and subjecting them to electrical and magnetic stimulation. The results showed that electrical stimulation significantly reduced neurite outgrowth in GelMA by 76%, while Gel-Amin mitigated this effect. Magnetic stimulation induced directional neurite outgrowth perpendicular to the magnetic field gradient in both hydrogels. SCs in Gel-Amin secreted less pro-inflammatory cytokines and angiogenic VEGF than in GelMA. MS in Gel-Amin led to the most favorable cytokine profile, supporting its use in immunomodulatory neural repair. This is one of the first direct comparisons of ES and MS in ionically conductive gelatin-based hydrogels, indicating Gel-Amin’s potential in neuroregeneration applications [62].

### 3.5. 3D Printed and Architecturally Tuned Conduits

Advanced production methods, including 3D bioprinting and digital light processing (DLP), have facilitated the development of intricate hydrogel structures. There are studies that employed DLP to manufacture conduits using a multichannel configuration that replicates the fascicular structure of peripheral nerves [63]. For instance, Lee et al. (2022) implemented microgrooved surface patterns on Poly(L-lactide-co-ε-caprolactone) membranes coated with gelatin to direct axon alignment [64], whereas Krieghoff et al. (2021) employed extrusion printing to fabricate modular gelatinous peptide-based conduits with adjustable degradation profiles [65]. To further mimic native nerve architecture, several groups employed 3D printing and microstructural refinement. In one such study Wu et al. (2023) [43] developed a conduit made by combining gelatin methacryloyl with silk fibroin glycidyl methacrylate (SF-MA), which are mixed and photo-crosslinked using digital light processing 3D printing. The hydrogels show improved mechanical strength, porosity, β-sheet crystallinity, and biodegradability. The G/F 1:1 composition provides the best balance between stiffness and biocompatibility. The study includes in vitro assays and in vivo trials using a 12 mm sciatic nerve defect model in rats. The conduit incorporates 7,8-dihydroxyflavone prodrug nanoassemblies, promoting axon elongation in PC12 cells. In vivo, it outperformed controls in nerve conduction velocity, myelin thickness, axon diameter, and muscle reinnervation, showing comparable results to autografts [43].

Furthermore, Maeng et al. (2023) have developed 3D printed hydrogels based on gelatin methacryloyl and polyethylene glycol diacrylate (PEGDA) for peripheral nerve regeneration [66]. The hydrogels were prepared using a 10% GelMA and 10% PEGDA formulation (G10P10), which showed enhanced mechanical strength, controlled biodegradability, shear-thinning and viscoelastic properties, and microporous and nanoporous wall structures. The study was conducted in vitro and in vivo using a rat sciatic nerve transection model with a 10 mm defect. The results showed improved sensory and motor recovery, enhanced muscle reinnervation, increased axon remyelination, and minimal inflammatory response in rats implanted with the gelMA/PEGDA multichannel conduit. These findings validate the biocompatibility and regenerative potential of the 3D-printed gelatin-based hydrogel nerve conduits [66]. In another study, the creation of multi-channel nerve guidance conduits containing Schwann cells using 3D bioprinting was investigated. A conduit made using methacrylate gelatin and N-(2-hydroxy)ethyl acrylamide (HEAA) for mechanical reinforcement was used. The conduit was then crosslinked by UV light, leaving high-precision microchannels inside. The gels have key properties such as high porosity, adjustable viscosity, enhanced mechanical properties with HEAA-bearing layers, and complete degradation of 5% GelMA in enzyme solution by day 11. The study used Schwann cells incorporated into the conduit, achieving high cell viability (>90%), uniform distribution around channels, and no observed cytotoxicity from HEAA layers [21].

In a study by Lee et al. (2022), a regenerative porous nerve guidance conduit was developed using poly L-lactide-co-ε-caprolactone and a swellable, microgrooved gelatin-based hydrogel fabricated via 3D printing [64]. The composite conduit integrates an electrospun PLCL membrane with a visible light-crosslinkable gelatin hydrogel prepared from gelatin type B, Ru(II)bpy_3_^2+^, and sodium persulfate. Crosslinking was achieved under blue light irradiation for one minute, inducing dityrosine bond formation. The hydrogel formed uniform microgrooved patterns on the flexible PLCL membrane, exhibited a swelling ratio of 209%, and maintained structural integrity for up to seven days. Its porous architecture facilitated nutrient diffusion and cellular migration, while also enabling sustained protein release for over 58 days through a mechanism governed by swelling and enzymatic degradation. In vitro, SH-SY5Y neuroblastoma cells cultured on the NGF-loaded hydrogel demonstrated enhanced adhesion, proliferation, and neurite outgrowth. In vivo, application of the PLCL/gelatin conduit, particularly when loaded with nerve growth factor, in a rat sciatic nerve defect model led to significant improvements in ankle angle recovery, muscle reinnervation, axonal diameter, myelin thickness, and nerve fiber density. Remarkably, the regenerative and functional outcomes were comparable to those achieved with autografts, supporting the clinical potential of this hydrogel-integrated conduit in peripheral nerve repair [64]. In another study by Krieghoff et al. (2021), a two-component hydrogel system was used, consisting of gelatinous peptides (GEL) crosslinked with anhydride-containing synthetic oligomers [65]. The formulations were mixed before extrusion-based 3D printing using a single-cartridge bioprinter. Variants of the oligomers and partial pre-derivatization were also tested. The mechanical strength of the hydrogels was manipulated by oligomer type and base, and the porosity was high and structurally regular. The constructs expanded 1.6 times in volume when hydrated, and showed both slow hydrolytic and rapid enzyme-mediated degradation. Tunable gelation kinetics allowed for an extended printability window of approximately 70 min. The results showed that the fabricated multi-channeled conduits were dimensionally stable during and after printing, confirmed cytocompatibility using hASCs and L929 fibroblasts, and successfully demonstrated aseptic printing. The hydrogel system is customizable for nerve guidance conduit applications and adaptable to various degradation profiles and biological cues [65].

**Figure 2:**
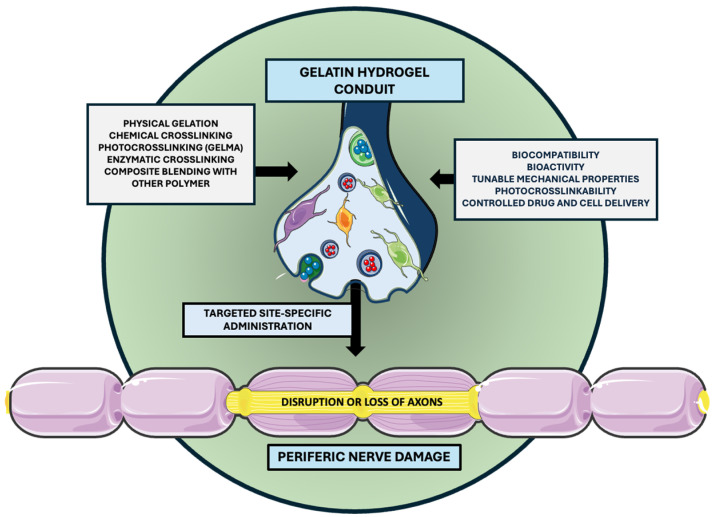
Localized delivery via multifunctional nerve guidance conduits. The selected artwork was taken or adapted from pictures provided by Servier Medical Art (Servier; https://smart.servier.com/, accessed on 13 March 2025), licensed under a Creative Commons Attribution 4.0 Unported License [33].

Additionally, the growing significance of integrating stimuli-responsive functionalities into 3D-printable hydrogel systems is remarkable. Localized stimuli such as pH, temperature, and mechanical stress play a critical role in guiding biological responses. To this end, a novel single-tailed surfactant (C14PMimBr) formed via dynamic imine bonding has been shown to self-assemble into micelles, vesicles, and hydrogels in aqueous solutions, enabling reversible structural changes under external stimuli and offering great promise for targeted drug delivery and intelligent material design [67]. In parallel, gradient polyelectrolyte membrane (GPM)-based hydrogels have been employed to fabricate self-powered ionic skins capable of detecting multiple environmental stimuli, pressure, temperature, and humidity, through mechano- and thermo-electric coupling, without requiring external power sources [68]. Advanced multifunctional nanoplatforms that combine photodynamic, photothermal, and chemodynamic therapies under a single-laser stimulus have also demonstrated efficient reactive oxygen species generation and potent in vivo tumor suppression while maintaining low off-target toxicity [69]. Such approaches may also be adapted for applications in peripheral nerve damage. Localized therapeutic delivery using multifunctional nerve guidance conduits designed to support regeneration at the site of peripheral nerve injury is depicted in Figure 2.

### 3.6. Stem Cell-Based and Neural Network Systems

Finally, several investigations have emphasized cell–matrix interactions and the potential of neural stem cells. Sugiyama made a study examining the impact of insulin-like growth factor 1 (IGF-1) on facial nerve function in guinea pigs has found that a gelatin-based hydrogel system can be used as a carrier for IGF-1 to treat peripheral facial nerve palsy. The hydrogel, MedGel, was applied topically to the compressed facial nerve in guinea pigs via an intratemporal surgical route. The hydrogel is known for its biocompatibility, biodegradability, sustained drug release, and ability to deliver bioactive proteins without systemic toxicity. The study used a guinea pig facial nerve compression model and confirmed IGF-1 receptor expression using qRT-PCR. Results showed that IGF-1-treated animals showed better eyelid closure and a 67% complete recovery rate compared to the saline control group. The study also found that IGF-1 receptor mRNA was present in the facial nerve and peaked 7 days post-injury, supporting receptor-mediated regeneration. This suggests that topical IGF-1 delivered via a gelatin hydrogel can enhance functional facial nerve recovery [70]. In another study, Aregueta-Robles investigates the role of miR-7 in modulating neural stem cell (NSC) behavior in a gelatin hydrogel conduit model for peripheral nerve repair. The hydrogel was used to encapsulate and transplant NSCs, providing structural support for nerve regeneration. The study found that the gelatin hydrogel functioned as a biocompatible scaffold for NSCs, supporting cell adhesion, migration, and bioactive molecule delivery. The effectiveness of the study was inferred from histological and functional outcomes, not from material characterization. The results showed that NSCs in gelatin conduits improved myelination, compound muscle action potential (CMAPs), SFI scores, and reduced muscle atrophy. However, miR-7 over-expression suppressed these benefits by inhibiting NSC migration/proliferation. Cdc42 was identified as a direct target of miR-7, mediating its effects. Functional recovery and histological markers were improved by NSCs but reversed when miR-7 was overexpressed. The study underscores the critical role of miR-7/cdc42 signaling in regulating NSC-driven regeneration within gelatin hydrogel conduits [57].

As mentioned previously, in a recent study, Esaki et al. explore the use of gelatin-based biosynthetic hydrogel (PVA-SG) to co-encapsulate Schwann cells and PC12 neuron-like cells for 3D neural network formation. The hydrogel was synthesized from poly(vinyl alcohol)-tyramine (PVA-Tyr) covalently crosslinked with sericin and gelatin (PVA-SG). Two formulations were prepared: 5 wt% (soft, ~2 kPa) and 10 wt% (stiff, ~16 kPa). The hydrogel showed tunable mechanical stiffness and was porous, degradable, and supportive of 3D cell encapsulation. It also supported ECM protein expression and better retained mechanical properties. The study was conducted in vitro, evaluating SC viability, morphology, ECM secretion, neurite outgrowth of PC12 cells, and co-culture behavior and network formation. Results showed that SCs in 10 wt% PVA-SG developed physiological morphologies, high viability, and ECM secretion. Co-encapsulation with PC12 cells promoted extensive neurite outgrowth, and SCs and PC12s formed 3D neural networks, mimicking in vivo architecture. The study highlights the importance of hydrogel stiffness in neural tissue engineering [56]. A comparative overview of recent preclinical studies utilizing gelatin-based hydrogels for peripheral nerve regeneration is summarized in Table 2.

## 4. Future Directions

The development of gelatin-based hydrogels as delivery systems for peripheral nerve repair is entering a pivotal stage, where the integration of cellular, molecular, and pharmacological components offers significant potential [71]. The studies analyzed in this review confirm that some gelatin hydrogels are highly biocompatible, tunable, and capable of incorporating diverse therapeutic agents, including Schwann cells, mesenchymal stem cells, growth factors, and bioactive small molecules [32]. Yet, a critical next step involves transitioning from proof-of-concept designs to translational studies that evaluate the localized delivery of clinically relevant drugs through these hydrogel matrices. While neurotrophic factors such as NGF, BDNF, and GDNF have long been established as key promoters of axonal regeneration, emerging evidence supports the therapeutic value of several pharmacological agents with antioxidant, neuroprotective, and metabolic-enhancing properties.

For instance, deproteinized hemoderivative of calf blood, has demonstrated significant efficacy in preclinical models of nerve injury by enhancing oxygen uptake, ATP synthesis, and neuronal survival [72]. Similarly, alpha-lipoic acid, a formulation combined with B-complex vitamins, addresses the reduction in oxidative stress while offering metabolic support for regenerating axons [73]. Alpha-lipoic acid has been shown to improve nerve conduction velocity and reduce reactive oxygen species in diabetic neuropathy models, while methylcobalamin enhances axonal transport, DNA synthesis, and myelin repair [74]. As a foundation for future hydrogel-based therapeutic strategies, we are currently conducting a preclinical study investigating the neuroregenerative potential of Calf Blood Hemodialysate and alpha-lipoic acid, administered systemically in a murine model of sciatic nerve injury. Adult male C57BL/6 mice were randomized into four groups: untreated controls, calf blood hemodialysate (C), alpha-lipoic acid (T), and combination therapy (C+T). The spared nerve injury model was employed to induce peripheral nerve damage. Treatment commenced 48 h post-injury, with Calf Blood Hemodialysate administered intraperitoneally for 10 consecutive days, and alpha-lipoic acid given at 20 mg/kg/day for 14 days. Functional recovery was assessed over 50 days using electromyography (EMG), the basso mouse scale for locomotion, and the beam walk test. Histological analyses focused on axonal density, myelin integrity, and inflammatory infiltration, while biochemical assays measured oxidative stress markers. Although most commonly administered systemically, the incorporation of these agents into hydrogel matrices represents a logical evolution. Treatment benefits could be amplified by local delivery reducing systemic exposure while maintaining high local bioavailability and sustained release at the injury site.

Additionally, natural compounds such as curcumin and resveratrol, both potent antioxidants with anti-inflammatory and pro-regenerative effects, could also be investigated within hydrogel-based platforms [75]. While their clinical translation has been limited by relatively low bioavailability, encapsulation within gelatin matrices may overcome these barriers as demonstrated in other organ systems where hydrogel formulations significantly enhanced local retention and bioactivity. Likewise, tacrolimus, a well-characterized immunosuppressant with powerful neurotrophic properties, has demonstrated dose-dependent improvements in axonal regeneration in animal models, particularly when delivered locally to minimize systemic toxicity [76]. Despite the individual promise of these agents, few studies have yet explored their co-delivery or comparative efficacy when embedded within biodegradable hydrogels. This represents a major opportunity for the field as researchers could design controlled preclinical studies that evaluate combinations of pharmacological agents and neurotrophic factors delivered via gelatin scaffolds. Additionally, standardized testing in chronic or large-gap nerve injury models, particularly in diabetic or aged animal systems, would provide much-needed insights into long-term outcomes.

Technological advances in hydrogel crosslinking, rheological tuning, and microfabrication also allow for the compartmentalization of multiple agents within a single construct, offering spatiotemporal control over drug release [77]. Such systems could be engineered to mirror the natural progression of nerve repair, from initial neuroprotection to later-stage axonal elongation and remyelination. A key priority for future studies will be to investigate the loading efficiency and release dynamics of neuroprotective and neuroregenerative compounds when encapsulated in GelMA or other gelatin derivatives, as these factors critically influence local drug concentrations and therapeutic windows. Moreover, as multi-agent strategies gain traction, it will be essential to elucidate the interactive effects of drug combinations and whether such combinations act synergistically or introduce unknown interferences.

Another important direction lies in the evaluation of functional recovery, not only through histological analysis but also via advanced behavioral and electrophysiological assessments. Moving beyond small-animal models, there is a pressing need for validation in large-animal models. Employing standardized and approved materials and real-world delivery protocols will be crucial in bridging the gap between laboratory innovation and clinical application.

### 4.1. Translational Studies

Despite encouraging preclinical evidence, the clinical translation of gelatin-based hydrogels for peripheral nerve injury remains largely unexplored. A targeted PubMed search using the terms “peripheral nerve” and “hydrogel” over the past 25 years yielded 689 entries, including 65 review articles and 3 systematic reviews, but no meta-analyses or clinical trials. Narrowing the search to “gelatin-based hydrogels” resulted in only 96 publications, with no systematic reviews or clinical studies being found. Interestingly, a search of the NIH ClinicalTrials.gov registry as of 20 May 2025, revealed no ongoing clinical trials involving hydrogel-based therapies for PNI. This lack of clinical data underscores the considerable gap between experimental advancements and their implementation in patient care. The challenges in translating promising hydrogel-based therapies for PNI into clinical practice are considerable. Key obstacles include optimizing drug release kinetics, ensuring long-term biocompatibility, and achieving consistent manufacturing quality and scalability. Moreover, current animal models often fail to capture the full complexity of human peripheral nerve injury, particularly regarding chronicity, comorbidities, and functional outcomes. Variability in experimental design, ranging from inconsistent outcome measures to differences in hydrogel formulations and delivery strategies, further complicates data interpretation and comparison across studies. Clinical acceptance is also delayed by regulatory obstacles and the lack of established criteria for hydrogel-based products. Although there are no clinical trials directly evaluating gelatin-based hydrogels in peripheral nerve regeneration, studies based on similar hydrogel-based scaffolds have begun to enter early clinical trials in other regenerative contexts [78].

### 4.2. Regulatory Considerations

Despite recent advances, regulatory hurdles remain significant. Currently, most gelatin-based hydrogels used in preclinical research are synthesized from animal-derived gelatin (e.g., porcine or bovine sources), which raises concerns about batch-to-batch variability, immunogenicity, and pathogen transmission [79]. To align with clinical standards, good-manufacturing-practice gelatin, ideally recombinant or plant-derived, must be adopted, and full traceability must be ensured. Additionally, photoinitiators such as Irgacure 2959, commonly used in GelMA synthesis, must be scrutinized for cytotoxicity and validated under biocompatibility protocols. For regulatory approval, hydrogel-based nerve conduits are likely to be evaluated as medical devices or combination products if drugs or biologics are co-delivered. Critical documentation will include device preclinical efficacy data in large-animal models, and long-term degradation and toxicology studies. Additionally, hydrogel sterility, post-sterilization mechanical integrity and endotoxin levels must meet stringent thresholds before clinical trials can be initiated. Collaborative networks between biomaterials scientists, clinical neurosurgeons, regulatory consultants, and industry representatives are essential to accelerate the translation of results.

## 5. The Evolving Role of Gelatin Hydrogels in Peripheral Nerve Repair

Hydrogels have emerged over the last decade as highly promising biomaterials in the context of peripheral nerve regeneration, offering the promise of a biologically relevant, tunable, and cost-effective alternative to traditional nerve repair methods such as autografts [71]. The studies analyzed in this review approach gelatin’s inherent biological characteristics, including its cell-adhesive motifs, enzymatic degradability, and structural compatibility with extracellular matrix components, significantly contributing to its success as a scaffold material. Additionally, gelatin’s capacity for chemical modification, particularly via methacrylation to form GelMA, has provided researchers with a platform to fine-tune key parameters such as stiffness, degradation rate, and bioactivity. Across both in vitro and in vivo models, gelatin hydrogels have supported the proliferation, migration, and differentiation of critical cellular mediators of nerve repair, including, neural stem cells, PC12 cells, and dorsal root ganglion neurons. Studies incorporating bioactive molecules, such as nerve growth factor, vascular endothelial growth factor, basic fibroblast growth factor, and 7,8-dihydroxyflavone (7,8-DHF), into gelatin hydrogels demonstrate their capacity to enhance regenerative microenvironments. Moreover, several studies have explored multi-modal enhancements that extend beyond biochemical support. The incorporation of conductive nanomaterials such as polyaniline/graphene and piezoelectric agents like ZnO into gelatin hydrogels introduces electrical conductivity and mechanoresponsive behaviors that mimic the electrophysiological properties of native nerve tissue. These features are particularly valuable given the importance of endogenous bioelectric cues in axonal pathfinding and synapse formation.

However, the current literature on conductive and electroactive gelatin systems remains largely confined to in vitro validation. The engineering of complex architectures using advanced fabrication techniques, especially extrusion-based 3D printing and digital light processing, has significantly expanded the utility of gelatin hydrogels in nerve conduit design. These technologies have enabled the creation of multichannel conduits that structurally resemble native peripheral nerves, thereby improving directional guidance for regenerating axons. Microgrooved and porous wall designs further enhance cell alignment, nutrient diffusion, and vascular integration. In particular, the use of visible-light crosslinking systems has opened new avenues for rapid, spatially controlled gelation without the risks associated with UV exposure. Despite the substantial progress shown in these studies, several critical issues remain unresolved. First, although gelatin hydrogels exhibit excellent short-term biocompatibility, their long-term stability and immunogenic profile are less well characterized, especially in large animal models. The rapid enzymatic degradation of unmodified gelatin can limit its usefulness in long-gap nerve injuries that require prolonged scaffold support. However, strategies such as increased crosslink density, and composite formulations dual-phase systems have shown promise in extending in vivo residence time, without compromising cellular compatibility [78,80,81].

Second, while the delivery of growth factors and stem cells within gelatin hydrogels has yielded encouraging results, the reproducibility of these effects is highly dependent on loading efficiency, release kinetics, and local tissue responses. The heterogeneity of gelatin sources, typically derived from porcine or bovine collagen, introduces batch-to-batch variability that complicates both experimental consistency and regulatory approval [82]. Purification, recombinant alternatives, or synthetic analogs may be needed for clinical translation. Another major challenge lies in scaling and standardizing the manufacturing process for clinical-grade gelatin hydrogels. Most current methods rely on lab-scale synthesis with custom crosslinking protocols and photoinitiator systems, which are not easily transferable to clinical trials. Furthermore, the field still lacks comprehensive guidelines on sterilization, storage, and long-term biosafety of gelatin-based nerve guidance conduits [83,84,85,86].

Importantly, there is also a significant gap between rodent studies and human clinical applications. Most reviewed studies use sciatic nerve models in mice or rats, which regenerate far more effectively than human peripheral nerves. Few studies have examined gelatin hydrogels in large animal models or under conditions that mimic chronic nerve degeneration, such as diabetic neuropathy or delayed nerve repair scenarios. These conditions are particularly relevant in the clinical context, where nerve injuries are often accompanied by comorbidities or prolonged denervation [87,88].

Finally, while many individual components of gelatin hydrogel systems, bioactive molecules, conductive additives, and microarchitectures have shown promise, their synergistic effects remain poorly explored. There is growing interest in developing “smart” nerve conduits that combine multiple functional domains: controlled release of trophic factors, dynamic mechanical responsiveness, immunomodulation, and even integrated biosensors for monitoring regeneration [89,90].

In summary, the incorporation of pharmacologically active compounds, such as the deproteinized hemoderivative of calf blood, alpha-lipoic acid, B-complex vitamins including methylcobalamin (vitamin B_12_), tacrolimus (FK506), curcumin, and resveratrol, into gelatin-based hydrogel systems is technically viable. These agents possess well-documented neuroprotective, anti-inflammatory, antioxidant, and neuroregenerative properties in preclinical models of peripheral nerve injury, effects that could be enhanced by local delivery via gelatin hydrogels. Future research should therefore leverage this advantage to engineer next-generation, multifunctional hydrogel-based nerve guidance conduits that integrate therapeutic efficacy with translational feasibility.

## 6. Conclusions

Gelatin-based hydrogels have emerged as one of the most promising biomaterial platforms in peripheral nerve tissue engineering due to their intrinsic biocompatibility, biodegradability, and physicochemical tunability. Derived from denatured collagen, gelatin closely mimics key components of the native extracellular matrix, providing a biologically supportive microenvironment for nerve regeneration. Numerous in vitro and in vivo studies have demonstrated that gelatin hydrogels facilitate essential regenerative processes, including Schwann cell adhesion and migration, axonal elongation, remyelination, and restoration of neuromuscular function. Chemical modifications, most notably methacrylation to produce gelatin methacryloyl, have enabled precise control over mechanical properties and network architecture via photo-crosslinking, resulting in structurally stable scaffolds suitable for neural applications. Functionalization of gelatin hydrogels with neurotrophic factors as well as small bioactive molecules and stem or glial cells, has transformed them from passive ECM mimics into bioresponsive platforms. Moreover, the integration of conductive nanomaterials and piezoelectric additives has allowed for electrical modulation of neural activity, addressing the electrophysiological demands of peripheral nerve tissues. The development of compartmentalized and stimuli-responsive hydrogel systems has further enhanced their potential for temporally controlled localized drug delivery. Advances in biofabrication techniques, particularly extrusion-based and digital light processing (DLP) 3D printing, now enable the creation of anatomically precise, multichannel nerve guidance conduits with tunable porosity, alignment cues, and spatially controlled distribution of cells and factors. Nonetheless, several challenges remain. Batch variability, limited long-term data, toxicity, degradation control, and scale-up for clinical-grade manufacturing continue to delay clinical trial translation.

## Figures and Tables

**Table 1:** Comparative analysis of gelatin-based hydrogels vs. other natural and synthetic hydrogels in peripheral nerve regeneration.

Parameter	Gelatin-Based Hydrogel	Collagen-Based Hydrogel	Chitosan-Based Hydrogel	Alginate-Based Hydrogel	PEG-Based Hydrogel
Biocompatibility	High; supports cell adhesion via RGD motifs	Excellent; native ECM protein; supports Schwann cells	Good; may require deacetylation to reduce immune response	Moderate; lacks native adhesion motifs without modification	High; non-immunogenic synthetic polymer
Biodegradability	Moderate; tunable via crosslinking (e.g., GelMA)	Rapid; enzymatic degradation	Moderate; adjustable via crosslinking	Fast; needs modification to delay degradation	Slow; highly stable unless modified
Mechanical Strength	Tunable; enhanced with GelMA or composites	Low; requires reinforcement	Good; suitable mechanical integrity in blends	Soft; mechanically weak without reinforcement	Tunable; wide range achievable with crosslinkers
Nerve Regeneration Efficiency	High; supports axonal growth and Schwann cell migration	High; proven in nerve guidance conduits	Moderate to high; effective especially in blends	Moderate; supports cell encapsulation, but limited axon guidance	Moderate; inert but functionalized PEG supports growth
Inflammatory Response	Low; minimal immune reaction	Low; generally well tolerated	Variable; mild to moderate inflammation possible	Low; but lacks immunomodulatory cues	Very low; highly inert and non-immunogenic
Drug/Growth Factor Delivery	Excellent; enables controlled delivery	Moderate; limited control over release kinetics	Good; functionalization improves loading	Poor; requires modification for sustained delivery	Excellent; ideal for controlled release systems
Ease of Fabrication and Modification	High; GelMA is photo-crosslinkable, printable	Moderate; limited crosslinking options	High; easily functionalized and blendable	Moderate; ionically crosslinked, easy to gel	Very high; synthetic control allows precise tuning
Clinical Translation Status	Preclinical; no clinical trials yet	Clinically used (e.g., Integra™) in soft tissue	Preclinical; some progress toward trials	Preclinical; limited nerve-specific applications	Used clinically in drug delivery; limited in nerve repair

**Table 2:** Overview of representative preclinical studies using gelatin-based hydrogels in peripheral nerve regeneration.

Study	Materials	Type	Model	Key Findings	Ref
Luo et al.	GelMA, bFGF, DPSCs	Photocrosslinked hydrogel	In vitro and 15-mm rat sciatic nerve model	Porous, bioactive, supports cell viability	[48]
Sukhinich et al.	Gelatin + glutaraldehyde	Simple gelatin conduit	In vivo mouse sciatic nerve	Good regrowth, no muscle atrophy reversal	[49]
Wu et al.	GelMA + SF-MA + DLP 3D printing	Prodrug nanoassemblies	12 mm rat sciatic nerve	Autograft-level results, improved myelination	[43]
Javidi et al.	Gelatin, PAG, ZnO, PCL/PVDF shell	Conductive, piezoelectric scaffold	PC12 cells	Enhanced neuronal differentiation	[61]
Maeng et al.	GelMA + PEGDA	3D-printed multi-channel	10 mm rat sciatic model	Good motor/sensory recovery, minimal inflammation	[66]
Zhang et al.	GelMA + HEAA + SCs	3D-bioprinted microchannel	In vitro and in vivo	High SC viability, strong mechanics	[21]
Zhang et al.	GM hydrogel + VEGF/NGF + chitin	Dual growth factor delivery	16-week rat model	Enhanced axon growth, angiogenesis	[58]
Lee et al.	PLCL + gelatin hydrogel + NGF	3D-printed porous conduit	Rat sciatic nerve defect	High protein release, comparable to autografts	[64]
Xu et al.	GelMA + VEGF165	Injectable hydrogel	Crush injury rat model	Sustained VEGF, functional and angiogenic gains	[20]
Li et al.	GelMA + CGRP	405 nm UV-crosslinked	Diabetic mouse model	Improved wound healing and anti-inflammatory	[59]
Krieghoff et al.	Gelatinous peptides + oligomers	3D-printable, tunable	Preclinical in vitro	Customizable, aseptic printing	[65]
Javanmardi	PA + Dex-Mp + HA	Injectable drug delivery	Rat crush injury model	Highest SFI, reduced vacuolation	[60]
Neuman	GelMA and Gel-Amin	Ionically conductive hydrogel	In vitro DRG, SCs	Magnetic stimulation improves outcomes	[62]
Sugiyama	MedGel + IGF-1	Topical hydrogel	Facial nerve guinea pig model	67% complete recovery	[70]
Zhou	Gelatin hydrogel + Neural Stem Cells (NSCs)	Gelatin hydrogel conduit	5 mm mouse sciatic nerve defect	NSCs promoted nerve repair; miR-7 inhibits NSC migration/proliferation via cdc42; overexpression worsened repair	[55]
Aregueta-Robles	Gelatin hydrogel + NSCs	miR-7 functional model	In vivo nerve injury	Improved CMAPs, reversed by miR-7	[57]
Esaki	PVA-Tyr + sericin + gelatin	3D co-culture hydrogel	In vitro PC12 and SCs	3D neural networks, stiffness tuned	[56]
Soucy et al.	GelMA + MeTro	Adhesive hydrogel	In vitro and ex vivo	Superior mechanical and adhesive properties	[45]

## Data Availability

Not applicable.

## Abbreviations

BDNF	Brain-Derived Neurotrophic Factor
BMS	Basso Mouse Scale
BWT	Beam Walk Test
CGRP	Calcitonin Gene-Related Peptide
DLP	Digital Light Processing
DRG	Dorsal Root Ganglia/Ganglion
ECM	Extracellular Matrix
ELISA	Enzyme-Linked Immunosorbent Assay
EMG	Electromyography
FKBPs	FK506-Binding Proteins
FR	Functional Recovery
GelMA	Gelatin Methacryloyl
HA	Hyaluronic Acid
IGF-1	Insulin-Like Growth Factor 1
LAP	Lithium Phenyl-2,4,6-Trimethylbenzoylphosphinate
MMP	Matrix Metalloproteinase
MSCs	Mesenchymal Stem Cells
MeTro	Methacrylated Tropoelastin
NGCs	Nerve Guidance Conduits
NGF	Nerve Growth Factor
NIH	National Institutes of Health
NSCs	Neural Stem Cells
PCL	Polycaprolactone
PEGDA	Polyethylene Glycol Diacrylate
PI	Photoinitiators
PNIs	Peripheral Nerve Injuries
PVDF	Polyvinylidene Fluoride
ROS	Reactive Oxygen Species
SCI	Spinal Cord Injury
SCs	Schwann Cells
VEGF	Vascular Endothelial Growth Factor
bFGF	Basic Fibroblast Growth Factor
